# Factors that influence clinical efficacy of live biotherapeutic products

**DOI:** 10.1186/s40001-021-00509-7

**Published:** 2021-05-04

**Authors:** Bruno Pot, Yvan Vandenplas

**Affiliations:** 1 Research Group of Industrial Microbiology and Food Biotechnology (IMDO), Department of Bioengineering Sciences (DBIT), Vrije Universiteit Brussel, Pleinlaan 2, 1050 Brussels, Belgium; 2 KidZ Health Castle, University Hospital Brussel, Brussels Health Campus, UZ Brussel, Vrije Universiteit Brussel, Laarbeeklaan 101, 1090 Brussels, Belgium; 3 Yakult Europe BV, Schutsluisweg 1, 1332 EN Almere, The Netherlands

**Keywords:** Administration mode, Dose, Matrix, Regulation, LBP, Probiotic, Quality

## Abstract

Traditional probiotics are increasingly being used in a medical context. The use of these products as drugs is considerably different from the traditional use as food or food supplements, as, obviously, the target population is different (diseased versus healthy or at risk population). Besides the target population, also the regulatory context is different, mainly with respect to production, administration regime and type of clinical studies required. In this paper we will, besides the regulatory differences, focus on aspects that may impact the efficacy of a live biotherapeutic product (drug), especially in a clinical setting. The impact of the dosage seems to depend on the strain and the application and may follow some rationale. In contrast, information on the impact of the time of administration or diet, is often still lacking. The matrix and the use of protective measures may clearly have an impact on the survival and efficacy of the strain.

## Highlights

- Probiotics and Live Biotherapeutic Products share common requirements, but differ in other aspects.
- Efficacy can be influenced by strain, dose, matrix, region, time of administration.
- Too few studies judge the impact of production-, quality- and administration parameters on LBP efficacy.

## Introduction

Probiotics are live microorganisms that, when administered in adequate amounts, confer a health benefit on the host [1]. However, the efficacy of probiotics depends on many variables and may be crucial for the expected outcome, especially when applied to patients. Therefore, it might be crucial to discriminate probiotics used as foods or food supplements from the live biotherapeutic products (LBPs) used in patients.

A registration as "drug" or "medical device" does not always guarantee quality of the product, as some products have a historical registration that today would no longer qualify when a new drug application would be made. Requirements for registration, moreover, can differ from country to country. From the regulatory perspective it is important to discriminate between a ‘health claim’, made for a food or a food supplement, and a ‘medical claim’, made for a drug. The difference is quite fundamental as a health claim, by definition, is addressing a healthy population. For regulatory authorities, clinical research on a population with suboptimal health or on an “at risk” population can also be considered a “healthy” population. Specific examples for this type of applications can, e.g., be antibiotic associated diarrhea (AAD), allergy, obesity, metabolic syndrome, frequency or severity of bacterial or viral infections, constipation, loss of bone mass, etc. In the case of a medical claim, the primary endpoint of the clinical trial should be linked to a specific disease, targeting patients with the intention to prevent, treat or cure disease. The intended use of the product, healthy vs sick individuals, will define the regulatory category in which the probiotic food or the LBP is to be registered.

Common to both registrations, however, is the requirement that the strain or strains in the product used for a study, needs to be properly identified at the genus, species and strain level [2]. According to the International Scientific Association of Pro- and Prebiotics [3, 4], a probiotic product label should not only disclose the genus, species and strain designation for all the strains in the product, but should also mention storage information, the best before date, the full ingredient list, the eventual presence of allergens, and should also provide intake recommendations including the clinical indication (formulated as a claim or not), the daily dosage and the information on the producing company. In both categories, drugs and foods, studies should be performed with the same dosage and composition present in the commercial product. According to the WHO/FAO guidelines for food trials, a good quality clinical study [randomized double-blind placebo controlled (RDBPC) trial] is required, while in general for drugs, at least two trials with the same primary endpoint [5–7] should be independently performed by two different centers or two multicenter trials.

### The importance of the dosage

The probiotic definition requires, in both food and drug settings, the administration of an 'adequate amount' in order to obtain the ‘probiotic’ or ‘medical’ benefit. However, it is not always obvious what an ‘adequate amount’ means, as the efficacy of the organisms administered might or might not be dose dependent.

In general, clinical studies with live microorganisms will mention the daily dose administered. In most cases, bacterial counts of live microorganisms are expressed as “Colony Forming Units” (CFU), although in 2015 the ISO 19344:2015 standard, also called IDF 232:2015, was described for the quantification by flow cytometry of lactic acid bacteria in milk and milk products, used as starter cultures, probiotics and fermented products. This method, however, is not yet used routinely, and even today most counts in the literature are still expressed as CFUs.

Although it seems logic to hypothesize "the more, the better", as suggested by the outcomes of some studies, the situation is not always that straightforward. Meta-analyses of numerous probiotic studies, with over 10 different products in AAD, confirmed earlier reports of a dose–response relation, showing that studies with 1 × 10^10^ CFU are generally not successful [8–10]. In contrast, for *Clostridioides difficile*-associated diarrhea (CDAD) no dose–response was observed [11–13]). For acute gastroenteritis in children, meta-analyses of 11 studies with a single strain, *Lacticaseibacillus rhamnosus* GG, also suggested a similar breakpoint of 1 × 10^10^ CFU [14]. While *Lc. rhamnosus* GG was effective at a daily dose of ≥10^10^ CFU and <10^10^ CFU, the latter dose produced results at the borderline of significance [14]). Unfortunately, only 3 of the studies used this lower concentration, making the analysis less reliable [14]. In addition, the *Lc. rhamnosus GG* strain is produced and commercialized by different companies, which may question the reliability of meta-analyses evaluating the efficacy of the same strain, produced by different companies.

While a preplanned sub-analysis of the use of lactobacilli for treating acute gastroenteritis suggested a dose–effect relationship [8], no such effects could be found for *Saccharomyces boulardii* on AAD [15–17], or *Limosilactobacillus reuteri* DSM 17938 on diarrhea [18]. Clearly, clinical dose–response trials should be set up with the intention to determine the lower non-effective dosages for different strains in different application fields.

A RDBPC trial in obese postmenopausal women with two doses of a multispecies LBP (1 × 10^9^ versus 1 × 10^10^ CFU ) showed a dose-dependent beneficial effect on cardiometabolic parameters and gut permeability [19]. A meta-analysis of LBP studies on blood pressure-lowering effects, concluded that doses higher than 10^11^ CFU were more effective than lower doses, while for other endpoints, such as necrotizing enterocolitis, prevention of atopic dermatitis and slow intestinal transit, no dose–response relation could be identified [20]. However, in a meta-analysis of 21 randomized controlled trials (RCTs) with LBPs used in a pharmacological therapy of patients with irritable bowel syndrome (IBS), the live microorganisms performed better than placebo in overall symptom response and quality of life assays, although were not significantly for individual IBS symptoms. Interestingly, single strains at a low dose, with a short treatment duration seemed more effective for both readouts [21]. These findings though, may need further confirmation. For at least two endpoints (quality of life and overall symptom response), the dose subgroup analysis was made with a cut-off value of <10^10^ CFU/day (LOW dose) and ≥10^10^ CFU/day (HIGH dose). Most likely, a more pronounced separation of both subgroups might allow to draw more reliable conclusions. As for the LBP type used, 5 single-strain- and 12-strain combinations were studied. The pooled relative risk (RR) in the single and combination subgroups was found to be 3.54 (95 % CI 1.48 to 8.45) and 1.41 (95 % CI 1.04 to 1.91), respectively, illustrating the high heterogeneity between the studies (*I*^2^ = 84.3 %, *p* < 0.001 and 68.5 %, *p* < 0.001) [21].

The dose results, however, matched earlier findings, presented in a meta-analysis of 64 studies with eight different gastrointestinal endpoints, in which the higher dose range (>10^11^ CFU) was not, while the lower doses were effective [22]. End-points such as immune markers, general health, and bowel function did not exhibit clear dose–response relations [20].

In conclusion, it can be stated that a higher dose might be worthwhile for AAD, as observed in both meta-analyses and dedicated dose–response studies, although these findings do not allow for extrapolation beyond the tested ranges [20] and it remains intriguing that for CDAD no similar dose–responses were observed. The lack of a clear dose–response for other endpoints does not mean this effect does not exist, but present data are insufficient to allow drawing firm conclusions [20]. More dedicated dose–response studies for different applications may be needed, especially using lower, non-effective doses (e.g., < 10^8^ CFU) [20]. The fact that most probiotic preparations on the market are foods or food supplements, with commercial benefits that are considerably lower than for (reimbursable) pharmaceutical drugs, might to a certain extent explain the overall lack of dosing studies. The optimal dosage, moreover, is likely to depend on the strain, on the indication and possibly on the endpoint. When dealing with live microorganisms, the observation that less than 100 bacterial cells of certain pathogens (e.g., *Shigella* or enterohemorrhagic *Escherichia coli*) can cause infection with serious consequences, illustrates that numbers may be less important than performance. Without any doubt, however, findings that higher dosages might result in a decreased efficacy warrant further research. It is in any case important for all clinical studies to clearly mention the dose and administration regime of the strain(s) used, of course always respecting the shelf life of the product. The dosage used in clinical trials (or the range thereof) should be the dosage mentioned in the regulatory dossier (whether food or drug) and should be the dosage made available in the product on the market.

### The impact of the time of administration and regional diet differences

The start of the LBP administration may also be determining its efficacy. As an example, the efficacy of an LBP in acute gastroenteritis has been related to an early administration [23]. Suez et al. administered for 4 weeks a previously unknown mixture of 11 strains to a group of 21 healthy human volunteers, *after* they were supplemented for 7 days with a broad-spectrum oral antibiotic mixture of ciprofloxacin (1 g/day) and metronidazole (1.5 g/day). Species-specific qPCR showed that in several stool samples 7 of the 11 administered species were significantly elevated from baseline, indicating that the administered strains may have been able to reach the lower part of the gastrointestinal tract of the host. In contrast to autologous FMT controls the LBP-consuming individuals, however, did not return to their baseline microbiome configuration for at least 5 months [24]. This is in sharp contrast to the generally accepted finding that chances to develop AAD are reduced or prevented by specific probiotic LBP administration. All in all these findings may actually confirm the results of a meta-analysis from Shen et al. [25], concluding that CDAD is reduced much more efficiently when the administration of the LBP is closer to the first dose of the antibiotic (reduction by > 50%). As in the Suez study [24] the LBP was not administered to human volunteers until 7 days after the start of the antibiotic treatment, the LBP may not have been efficient enough to restore the microbiota, heavily damaged by the broad-spectrum antibiotic. In this particular case the timing seems very important, intuitively suggesting that when an LBP is administered along with the antibiotic, it may prevent, possibly by mechanisms of competitive exclusion, large-scale dysbiosis and overgrowth by opportunistic pathogens.

The rationale related to administration regime and timing may therefore depend on the anticipated mode of action (MoA) of the administered strain. The MoA can be microbiological in nature (e.g., competitive exclusion), metabolic (e.g., cross-feeding with other members of the microbiota or production of antimicrobial metabolites), immunological [e.g., stimulation of natural killer (NK) cells, secretarial IgA or regulatory T cells (Treg)] or physiological (e.g., production of short chain fatty acids or impact on bile acid metabolism). In most of these mechanisms, a timely administration seems warranted.

Recent works suggests that the geographical region where a study is performed may also be one of the factors influencing the therapeutic or prophylactic outcome of an intervention. A systematic review with meta-analysis of *Lc. rhamnosus* GG in the treatment of acute gastroenteritis in children, seemed to have been more effective when used in European countries as compared to non-European countries [14]). In contrast to the administration timing discussed above, a clear mechanistic explanation of this observation is currently not available, although a geography-driven difference in microbiota composition might be a likely factor. The latter can be influenced by diet, rich or poor in, e.g., microbiota-influencing factors (fermented food products, fiber-rich foods, prebiotics, etc.). The consumption of medication such as antibiotics and proton pump inhibitors may also impact the microbiota composition [26]. The impact of drugs on the microbiota was recently reviewed by Vila et al. [27]. Since the consumption of these drugs differs from region to region, this may in part explain the above observation.

### The influence of the matrix

It is generally accepted that the survival of LBPs, and therefore their clinical efficacy, depends on manufacturing and storage conditions, the matrix and the physicochemical properties and intrinsic resistance of the strains to these environmental stresses [28]. The matrix consists most of the time of (skimmed) milk and/or carbohydrates. The matrix in which the probiotic or LBP is preserved and administered, is increasingly being studied and is now well documented for its impact on, e.g., the strain viability, the product shelf life and the survivability of the strain after intake. The impact of the matrix on viability may be completely different from the effect of the matrix on the in vivo efficacy. A number of recent papers highlighted the effect of the carrier matrices and storage conditions on the quality of probiotic or LBP [29]. The importance of the matrix has been mainly studied in (fresh) food products and may be less well known for LBPs or food supplements.

#### Influence on survival

Loss of viability of the strain(s) starts already at the end of the production process. Also, products claiming to conserve the same number of live bacteria may contain numerous undetected dead bacteria, which may be biologically relevant, as intact bacteria may, e.g., still interact with the immune system. Matrix components, such as fats, proteins, carbohydrates, additives and flavoring agents have been shown to alter probiotic efficacy and viability [29]. The strong interaction between the strain and the matrix has been studied mainly for fresh food products with probiotic strains. The effect of different matrices on bacterial resistance to in vitro-simulated gastrointestinal conditions was demonstrated by Casarotti and Penna [30]. The viability of bifidobacteria and lactobacilli differed in a specific cheese matrix [31]. In a very interesting study, Rodrigues et al. [32] compared the effect on survival, metabolism and cell integrity before and after simulated digestion of preparations containing inulin and *Lactobacillus acidophilus* LA-5 and *Bifidobacterium animalis* subsp*. lactis* BB12 in equal amounts. The carrier matrices compared were fermented milk, ice cream and a dry dietary supplement. The survival of both strains was evaluated on agar plates with MRS (total count) or modified MRS (different for lactobacilli and bifidobacteria) and by flow cytometry. Flow cytometry was used to measure the metabolic status (live, damaged, dead), scanning electronic microscopy to measure cell morphology. Fermented milk was found to be significantly better (*p* < 0.05) in preserving viability, before and after the digestion process, measured both by plate counting and flow cytometry. Both dairy products also preserved cell integrity better than the dry supplement. The authors argue that the presence of large amounts of total solid milk components (including milk fat) may improve bacterial resistance towards the acid environment of the stomach and physically protect bacteria against enzyme action in the stomach and small intestine [33–35] and could thereby help to maintain the metabolic activity of the microbial cells [36].

Saxelin et al. [37], in a randomized, parallel-group, open-label trial studied oral and fecal recovery before, during and after administration of a combination of *Lc. rhamnosus* GG and LC705, *Propionibacterium freudenreichii* subsp. *shermanii* JS, and *B. animalis* subsp. *lactis* Bb-12, as capsules, or in a yogurt or cheese matrix. They found no significant influence on the fecal recovery of the lactobacilli, but the matrix did affect the fecal counts of propionibacteria and bifidobacteria, which were lower when consumed through the cheese matrix (*p* < 0.01 and *p* < 0.001, respectively). The latter organisms were found to be supported best by a yogurt matrix, in which they also had the longest recovery times after ceasing the intervention (*p* < 0.05 for both strains). In general, the administration matrix did not influence the recovery times of the lactobacilli. The authors concluded that the consumption of probiotics in yogurt matrix is highly suitable for studying potential health benefits and capsules provide a comparable means of administration when the viability of the strain in the capsule product is confirmed.

Loss of “viability” in the stomach is not necessarily affecting the efficacy of a probiotic preparation in a fatal way. Bedani et al. [38] used an in vitro-simulated digestion protocol to compare fermented soy product formulations. The authors noticed that strain *B. animalis* subsp*. lactis* Bb-12 was better preserved in the food matrix, as compared with the fresh culture. They also observed an increased viability of both strains LA-5 and Bb-12 after the enteric II phase, showing that some damaged cells were able to resuscitate and multiply. Rodrigues et al. [32] hypothesized that the higher metabolic activity of the bacterial cultures in fermented milk could be due to several factors, including (i) the fermentation step, e.g., with adaptation to acid stress, (ii) the shorter shelf life of the fresh products, (iii) the higher water activity and, (iv) the lower storage temperature, increasing the survival in fermented milk as compared to ice creams or dietary supplements.

It has been hypothesized that in individual strains, different stress response mechanisms could be involved, notably transport systems, proton pumps, DNA chaperones and other cellular processes involved in cell integrity maintenance or in energy metabolism, and therefore in, e.g., acid and bile tolerance [39–44]. These mechanisms are clearly not limited to the strains mentioned above. Sagheddu et al. [45] investigated two *Lc. rhamnosus* strains in different food matrices (carrot juice, rice cream and cow’s milk, fermented or not) for their ability to survive in vitro treatment with four different simulated gastric juices. Rice cream was found to be less protective to simulated gastric juices than fermented carrot juice or milk, differences that may be linked to the strain. The authors suggested that the significant strain-differences between the two strains of *Lc. rhamnosus* tested [LMG S-29885 and ATCC 53103 (= strain *Lc. rhamnosus* GG)] could be attributed to the environmental origin of LMG-S-29885, isolated from weeds, in contrast to strain ATCC 53103, isolated from the intestinal tract of a healthy human being. They hypothesize that strain LMG S-29885 may have developed adaptations to a vegetable matrix and therefore to more harsh conditions.

Other non-dairy food matrices have recently been compared for their ability to support viability and extent the shelf life of probiotic products, such as cereals, pulses, or mixtures thereof, vegetables, fruits, or combinations thereof and unconventional foods [46]. Not unexpectedly, the shelf life of these products varied from 7 days to 4 weeks and depended on the initial count, the temperature, as well as on the strain and the matrix. Soymilk, as an example, was the most promising substrate among the pulse-based ones and cereal-based products were better than fruits in terms of probiotic count, nutritional properties, acceptability and fermentation time [28].

#### Influence on clinical endpoints

While studies that compare the effect of variable matrices on clinical efficacy are scarce, some in vivo studies do show strain-dependent matrix effects on the GIT survival of probiotic bacteria [28, 29]. Available clinical studies mostly compare identical matrices, containing different probiotic content. Only a few studies explore the effect of the matrix itself.

A comparison between lactose-hydrolyzed low-fat milk (10^10^–10^11^ CFU/day) and freeze-dried powder (10^10^–10^11^ CFU/day), showed that both products significantly reduced the duration of the diarrhea by approximately 1 day, as compared to a control, but no matrix-specific effects were found [47]. Low-fat milk (2.5 × 10^10^ CFU/day) versus lactose-hydrolyzed low-fat milk (2.5 × 10^10^ CFU/day) favored the lactose-hydrolyzed low-fat milk, showing a higher NK cell activity [48, 49]. However, clinical impact was not evaluated in these studies. Similar patterns of immune stimulation were observed when studying the impact on the upper respiratory tract of healthy adults of a *Bifidobacterium* strain, whether administered in yogurt or through capsules [50]. The effect of cheese (delivering 1 × 10^10^ CFU/day of the strain *Lactiplantibacillus plantarum* TENSIA) versus yogurt (6 × 10^9^ CFU/day of the same strain) was compared for an effect on blood pressure, among other parameters [51]. Findings were that the diastolic blood pressure was significantly improved for the cheese and yogurt matrix (*p* < 0.005), but the systolic blood pressure reduction from baseline was only significant for the cheese matrix (*p* < 0.005), although a strong trend was also found in the yogurt matrix (*p* = 0.055). However, participants in the cheese group consumed a larger daily dose (1 × 10^10^ CFU/day) of the strain versus the yogurt group (6 × 10^9^ CFU/day), which might explain the difference observed [51].

In vitro, the anti-pathogen effect of strain *Lc. rhamnosus* GG against outbreak-causing serotypes of *Shigella dysenteriae* (ATCC 29026) and *Shigella flexneri* (ATCC 12022) differed according to the matrix. Comparing an apple-based with a sea buckthorn-based beverage matrix, fortified with 5% malt extract powder, showed 99% clearance of both pathogens within the first hour for the latter product, while only 11% of *Sh. dysenteriae* and 5.6% of *Sh. flexneri* by the apple beverage [52]. The authors, however, also showed by PCA analysis that the total phenolic content of the matrices may have influenced the efficiency of the products to kill the pathogens [52]. Clearly, the endpoint measured may be affected by the matrix composition, on top of or at the expense of the active strain. Clinical studies therefore should preferably be performed with the final commercial product.

#### Protective measures (encapsulation)

Some interventions, such as microencapsulation can not only provide increased stability during storage, but may also beneficially affect the viability throughout the passage of the upper gastrointestinal tract [28]. The selection of an adapted dehydration method, a protectant agent, as well as storage conditions may therefore be crucial to an optimal preservation, delivery and efficacy. Depending on the type of encapsulation used, the strain may be released after the passage of the stomach or, much later, at the end of the small intestine [53].

In vitro predictive gastrointestinal models are very helpful and reliable to study these parameters and may assist in the development of new galenical formula with probiotics. Venema et al. achieved to deliver >10-fold higher numbers of viable cells to the small intestine by the use of an enteric-coated tablet [54]. The three probiotic strains concerned, *Lactobacillus gasseri* PA 16/8, *B. longum* SP 07/3 and *B. bifidum* MF 20/5 were evaluated in a validated artificial in vitro model of the stomach or the stomach and small intestine. Survival of the strains in an unformulated powder was improved from 5.3% and 2% for bifidobacteria and 1% and 0.1% for *L. gasseri* (survival after transit through the gastric compartment, respectively, the complete gastrointestinal tract) to 72% (gastric survival) for bifidobacteria, and 53% (gastric survival) for *L. gasseri*. This optimization also increased the small intestine survival by about an order of magnitude. The new galenical formulation was finally tested in the TIM-1 model, modeling the stomach and small intestine, which showed a survival of 13.5% for bifidobacteria under dynamic conditions simulating elderly and 7.3% under physiological parameters simulating adults.

The risk of interferences with (small) variations in production and storage might make this a difficult topic for reliable research. Grześkowiak et al. [55] recovered *Lc. rhamnosus* GG isolates from more than 13 different probiotic foods and food supplements, obtained from different countries, and compared their in vitro anti-pathogenic abilities with the original *Lc. rhamnosus* GG isolate from S. Gorbach. Isolates, shown by different typing techniques to be identical to *Lc. rhamnosus* GG (ATCC 53103), were isolated from capsule products (four isolates), from commercial infant foods (two isolates), from freeze-dried powders (three isolates) and from soft agar (four isolates). All *Lc. rhamnosus* strains were able to inhibit the adhesion to colonic mucus of both Gram-positive and Gram-negative pathogens [*Cronobacter sakazakii* (ATCC 29544), *Staphylococcus aureus* (DSM 20231), *Clostridium perfringens* (DSM 756) and *Salmonella enterica* serovar Typhimurium (ATCC 12028)], albeit at considerably different levels [55]. Quantitative adhesion differences were measured for *C. sakazakii*, *S. enterica* serovar Typhimurium and *C. perfringens* (*p* < 0.001) but not for *S. aureus* (*p* < 0.352). While the probiotic capacity to inhibit pathogens was present in all products, original properties may be impacted by the industrial production processes, or as argued above, the food matrix used.

### Regulatory importance

Medical as well as health claim regulations require that probiotic effects are demonstrated with the final product, whether it is a food, a food supplement or a drug. This might seem defendable, given the examples of the impact of the matrix described above, but may not be logical in comparison to the absence of efficacy studies of traditional drugs in relation to different dietary habits, administration modes or microbiota composition, which were extensively shown to impact on drug efficacy [56], a phenomenon also known as pharmacomicrobiomics [57].

The requirements may not only be challenging for the development of probiotics or the establishment of probiotic efficacy, but the need for repeated and extensive human clinical trials in absence of reimbursement schemes, may be economically difficult and even raise ethical concerns by withholding effective and safe treatment possibilities longer than needed [58].

Fact is that human clinical trials comparing different matrices with a clear health endpoint are scarce and additional clinical studies could help to alleviate the current uncertainty. In absence of sufficient clinical support, a scientific rationale to support the bio-equivalency of different matrices may help to circumvent the requirement to re-conduct expensive human clinical trials on probiotics delivered in new matrices [58].

In conclusion, while the matrix can obviously have an impact on the viability of the probiotic product before intake as well as on its survival in the host, there are very few data on the exclusive impact of the matrix on the clinical outcome. According to the limited data available, a dairy matrix often seems to offer the best survival conditions. This may have an impact in individuals on milk-free diets [45]. Intake of freeze-dried or otherwise processed food supplements or drugs is almost unavoidably linked to food intake. Therefore, information linked to food should be considered as relevant for future clinical studies, steering the selection of the most suitable probiotic strain as well as defining the optimal administration regime. A final remark concerns the fact that, depending on the mode of action of the strain(s) used, some applications will not require live microorganisms. It can be expected that dead but intact cells, so-called paraprobiotics, can interact with, e.g., the immune system. According to the FAO/WHO definition, however, dead cells cannot be called probiotic.

### Quality and adverse effects

An ESPGHAN commentary focused on the lack of quality control during the production of the majority of the commercial probiotics (almost exclusively foods) used in the clinic [59]. For LBP manufacturing, an intricate production process is required that ensures both high yield and stability and must also be able to meet requirements such as the absence of specific allergens, which precludes some obvious culture media ingredients [60]. Reproducibility is important to ensure constant high performance and quality [60]. To ensure this, quality control throughout the whole production process, from raw materials to the final product, is essential, as is the documentation of this quality control. Pharmaceutical product formulation requires extensive skills and experience. Traditionally, probiotic lactic acid bacteria and bifidobacteria have been incorporated in fermented dairy products, with limited shelf life and refrigerated storage. Currently, probiotics may be incorporated in dietary supplements and other "dry" food matrices which are expected to have up to 24 months of stability at ambient temperature and humidity [60]. In the case of pharmaceutical applications, companies should undergo third-party evaluations to certify LBP quality and label accuracy [61], since these products can be used in critically ill patients. While probiotics are generally considered safe and adverse events of probiotics are rare and almost always reversible, the problem may arise from contamination during manufacture, processing or storage.

For probiotics in fresh food products, the normal hazard analysis rules by control of critical points (HACCP) will apply. For LBPs in a pharmaceutical setting, production conditions should comply with pharmaceutical GMP (good manufacturing practice) conditions. In the latter situation, proper microbiological controls should be performed, using molecular and sequencing technology to detect the presence of contaminants or genes which could be considered harmful for the patient. In the case of clinically validated multi-strain preparations, care should also be given to the ratio of the different strains (resembling the ratio of the study product), taking into account their potential difference in viability over time.

Up to a certain limit, adverse effects of LBPs may be found acceptable when administered in some life threatening indications, such as necrotizing enterocolitis, but only if analyses show a positive benefit/risk ratio. In the past, some adverse effects have been associated with probiotic administration. The most well-known case is the Dutch PROPATRIA study in which probiotics were used in the prophylaxis of severe pancreatitis [62]. The study had to be stopped early because of the high incidence of adverse effects [63]. Later analyses of the cases showed that the LBP strains had not been the cause of the increased mortality, but a combination of proteolytic pancreas enzymes in combination with high in situ delivered levels of lactic acid producing bacteria together with high levels of carbohydrates from enteral nutrition leading to local fermentation in an already ischemic gut [64, 65]. In another example, neonatal sepsis has been associated to *Lactobacillus* administration [66, 67]. Whole-genome-based sequencing showed that lactobacilli, isolated from the patients' blood were identical to the lactobacilli of the LBP used [66], suggesting an increased risk of *Lactobacillus* bacteremia in intensive care unit (ICU) patients treated with LBPs as compared to those not treated. Further analysis, however, has shown that the colonization of a catheter may have been the source of the direct transmission of the LBP to the bloodstream [66]. In another case, the role of an indwelling vascular catheter, contaminated air, environmental surfaces and hands, following the opening of a packet of *S. boulardii* was suggested as the cause of fungemia in ICU patients [68].

Daniel et al. [69] compared the translocation potential of strains from food and infection sources by marking them, for tracking purposes, with known antibiotic resistances before testing the strains for their translocation potential. When administered to healthy mice none of the strains translocated. When administered to animals with a damaged barrier, due to the administration of TNBS, only strains from infection sources were found to translocate to the different organs in the mice, while food-derived strains did not. This experiment illustrates the strain specificity of the translocation potential as well as the importance of the host’s status and indicates that translocation potential should be considered as an important safety characteristics when developing LBPs [70].

The fact that currently most probiotics on the market today have a food status (and not a drug status) is not promoting the follow-up of reported side effects. While adverse events during clinical trials with foods and drugs are generally monitored, the nutrivigilance, in analogy with pharmacovigilance after the introduction to the market, is not yet very well developed for foods.

Reports on adverse effects of living microorganisms may stimulate research on the mode of action, opening the option to replace the complete live microorganism by thermally or otherwise killed microorganisms or their metabolites. This postbiotic or paraprobiotic concept may resemble much more the traditional drug approach and can eliminate the need for extensive microbiological testing. A nice example of this approach could be the peptidoglycan and derived muropeptides which could be identified as the active compounds of anti-inflammatory functionality and which were suggested to represent a useful therapeutic strategy for IBD,as suggested by Elise Macho-Fernandez et al. [71, 72].

Establishing validated methodologies for all aspects of quality assessment is an essential component of this process and can be facilitated by established organizations. Emerging methodologies including whole genome sequencing and flow cytometry are poised to play important roles in these processes [61].

### The regulation: health versus medical claims

Health claim substantiation for probiotics, in most countries, follows a separate regulation, different from the traditional clinical drug registration procedures for LBPs. In the USA, the FDA is in charge of both types of registrations, in Europe food related health claims are evaluated by the European Food Safety Authority (EFSA), while medical-related claims are treated by the European Medicine Agency (EMA). In both cases, the exact procedures to follow are not very well described. EFSA has published some guidelines: general [73, 74]; safety related [75, 76]; or functionality related [77, 78], but the outcome of a dossier remains uncertain and depends on an evaluation by the Panel on Nutrition, Novel Foods and Food Allergens (NDA panel), composed of mostly external experts. In order to clarify the situation, the International Probiotic Association Europe (IPA-EU; [79]) in January 2019 had a meeting with EFSA to discuss the current situation [80, 81] and explore, in analogy with other parts of the world, how to improve the possibilities of probiotic producers to communicate about the possible health benefits of their products: “Probiotic” as a generic descriptor or a food category, the “nutrition claim” option, or the option of a “positive list”, like it exists in Canada [82]. At the EMA level there is currently also no ready-to-use protocol for the submission of a LBP drug dossier, as EMA has so far not dealt with live microorganisms and the difficulties this may bring, as discussed above. The Pharmabiotic Research Institute (PRI, [83]) has been in contact with the EMA to develop such a protocol. Some elements of such a dossier, like standardized enumeration by flow cytometry of live, dead and active bacteria [84] have in the meantime been standardized, ISO certified and taken up in the European Pharmacopeia [85]. The hope therefore is that in the coming years a number of dossiers will be submitted to the EMA, allowing to further develop, evaluate and improve current procedures and to come to a harmonized European set of instructions for the evaluation of LBP-based drug dossiers. In the absence of such a formal regulation, one can expect to see local, national initiatives such as the one recently published in the Netherlands on the use of the term probiotic [43]. According to ISAPP, a probiotic product label should disclose the genus, species and strain designation for all strains in the product; ingredients/allergens; claims/recommended use; daily dosage; storage information; best before date; company name/contact information. These specifications, however, might need to be extended for a *drug product* containing live microorganisms.

## General conclusion

The use of LBPs to prevent or cure disease is increasing, especially in application areas where traditional pharmaceutical approaches do not yield optimal results or when long-term side effects make a long-term treatment period with traditional drugs difficult. The registration of LBPS as pharmaceutical drug, however, is rather rare. The use of live microorganisms in potentially vulnerable populations is different from their use as food or food supplements in a healthy population. Besides the target population, the requirements for clinical support, manufacturing conditions, administration regimes and adverse event monitoring may differ.

Moreover, clinical studies with live microorganisms differ considerably from clinical studies with traditional molecular drugs. The effectiveness of preparations with live microorganisms may depend on viability of the preparation (dose), which itself may depend on the type of matrix, the manufacturing and packaging conditions, the use of encapsulation technology and the mode of administration, e.g., interference with the environment (microbiome composition or diet). For most of these factors, there is very little systematic information available. The information is, moreover, disperse, as results most likely may differ from application to application and from strain to strain. The latter is most often the conclusion of systematic and Cochrane reviews. Standardization, while desirable, is more difficult than for traditional drugs in the light of this viability issue. Issues related to diet and geographical differences, may also play.

Therefore, there remains a clear need for further studies that determine the impact on viability of the production parameters, storage matrix and storage conditions on individual strains. For each application, moreover, dose–response studies should be performed, preferably in different environmental conditions and diets and with different administration regimes. While this will unavoidably increase the development cost, it will also improve the acceptability of this type of drug in the medical community and discriminate the use of probiotics as drugs from its use as food.

## Data Availability

Not applicable.
